# Heart rate reduction with esmolol in septic shock: effects on microcirculation

**DOI:** 10.1186/cc10809

**Published:** 2012-03-20

**Authors:** A Morelli, A Donati, A Di Russo, F D'Ippolito, A Carsetti, R Domizi, A D'Egidio, C Raffone, C Scarcella, C Ertmer, S Rehberg, P Pietropaoli, M Westphal

**Affiliations:** 1 University La Sapienza, Rome, Italy; 2 Marche Polytechnique University, Ancona, Italy; 3 University Hospital of Muenster, Germany

## Introduction

Preclinical and clinical studies report that β-blockers may be an interesting option to attenuate the deleterious effects of prolonged catecholamine exposure during septic shock. Nevertheless, there are concerns that β-blockers may have negative chronotropic and inotropic effects leading to inappropriately low cardiac output. The objective of the present study was therefore to elucidate whether a reduction in heart rate (HR) with esmolol may negatively affect microcirculation in patients with septic shock who remained tachycardic after hemodynamic optimization.

## Methods

After 36 hours of initial hemodynamic stabilization, 11 septic shock patients with HR >95 bpm and requiring norepinephrine (NE) to maintain mean arterial pressure (MAP) between 65 and 75 mmHg, despite adequate volume resuscitation, received a continuous esmolol infusion to maintain HR between 94 and 80 bpm. NE was titrated to achieve a MAP between 65 and 75 mmHg. Data from right heart catheterization and sidestream dark-field imaging were obtained at baseline and after 24 hours.

## Results

Apart from a statistically significant decrease in HR and cardiac index (CI) (*P *< 0.05), stroke volume (SV), microvascular flow index of the small vessels (MFIs) and norepinephrine requirements did not vary during the 24-hour observational period. Results are summarized in Table 1.

**Table 1:** 

	Baseline	24 hours
HR	119 ± 12	85 ± 9*
CI	4.4 ± 1	3.1 ± 1*
SV	81 ± 35	80 ± 23
MFIs	2.6 ± 0.6	2.8 ± 0.3
NE	0.7 ± 0.7	0.5 ± 0.4

*P < 0.05.

## Conclusion

In patients with established septic shock who remained tachycardic after hemodynamic optimization in accordance with the current guidelines, titration of esmolol to reduce the HR to a predefined threshold did not affect microcirculatory blood flow.

